# Strengthening antimicrobial stewardship in public health facilities in Malawi through a participatory epidemiology approach

**DOI:** 10.1093/jacamr/dlaf103

**Published:** 2025-06-11

**Authors:** Adriano F Lubanga, Akim N Bwanali, Sibongile Kondowe, Ellen Nzima, Abgail Masi, Yaleka Njikho, Cynthia Chitule, Gracian Harawa, Steward Mudenda, Gillian Mwale, Tumaini Makole, Samuel Mpinganjira, Thomas Nyirenda, Collins Mitambo

**Affiliations:** Research & Development, Clinical Research Education and Management Services (CREAMS), Lilongwe, Malawi; Research & Development, Clinical Research Education and Management Services (CREAMS), Lilongwe, Malawi; Ministry of Health, Antimicrobial Resistance National Coordinating Centre (AMRCC), Lilongwe, Malawi; Ministry of Health, Antimicrobial Resistance National Coordinating Centre (AMRCC), Lilongwe, Malawi; Ministry of Health, Antimicrobial Resistance National Coordinating Centre (AMRCC), Lilongwe, Malawi; Ministry of Health, Antimicrobial Resistance National Coordinating Centre (AMRCC), Lilongwe, Malawi; Research & Development, Clinical Research Education and Management Services (CREAMS), Lilongwe, Malawi; Ministry of Health, Antimicrobial Resistance National Coordinating Centre (AMRCC), Lilongwe, Malawi; Department of Pharmacy, School of Health Sciences, University of Zambia, Lusaka, Zambia; Antimicrobial Resistance Coordinating Committee, Zambia National Public Health Institute, Lusaka, Zambia; Department of Obstetrics and Gynecology, Queen Elizabeth Central Hospital, Blantyre, Malawi; Department of Pharmacy, Pharmacy Council of Tanzania, Dar Es Salaam, Tanzania; Department of Global Health, University of Washington, Seattle Campus, Seattle, WA, USA; Research & Development, Clinical Research Education and Management Services (CREAMS), Lilongwe, Malawi; Strategic Partnerships and Capacity Development– Head of Africa Office, European and Developing Countries Clinical Trials Partnership (EDCTP), Cape Town, Republic of South Africa; Department of Global Health, Stellenbosch University, Stellenbosch, Republic of South Africa; Ministry of Health, Antimicrobial Resistance National Coordinating Centre (AMRCC), Lilongwe, Malawi

## Abstract

**Background:**

In healthcare settings, antimicrobial resistance (AMR) is largely driven by excessive use of antibiotics. Empirical prescription largely remains common due to fragile healthcare systems characterized by lack of appropriate diagnostic services. Despite limited data on the epidemiology and the burden of AMR due to the scarcity of routine microbiology facilities, it is evident that Malawi shares a heavy burden of AMR. Effectively implemented antimicrobial stewardship programmes have demonstrated successes in minimizing inappropriate antibiotic usage, and curbing the burden of AMR. However, there are limited data on how antimicrobial stewardship teams can effectively deliver their roles in hospital settings in resource limited settings, including in Malawi.

**Methods:**

Malawi’s Antimicrobial Resistance National Coordinating Centre (AMRCC) in collaboration with Clinical Research Education and Management Services (CREAMS) conducted participatory workshops with hospital-based antimicrobial stewardship committees aimed at establishing drivers of resistance and antibiotic overuse in hospitals from the perspective of the committees, and co-design facility-friendly intervention against AMR. The workshops consisted of participatory discussion, sorting and design thinking exercises, utilizing principles of implementation research. All the interviews were recorded, transcribed and thematically analysed, revealing key drivers for antibiotic overuse and resistance in hospital settings. Data were analysed using thematic content analysis.

**Results:**

Key drivers of AMR included limited antibiotic formulary access, poor cross-sectoral coordination challenges between healthcare, veterinary services, government agencies and private facilities, and culturally specific barriers. The participants recommended regular training for healthcare workers on AMR and infection prevention and control (IPC), widespread dissemination of AMR findings, public awareness, introducing electronic monitoring systems and the enforcement of antibiotic restriction policies as the best measures for improving rational antimicrobial use and controlling the spread of AMR.

**Conclusions:**

Our findings underscore the complexity of the drivers for antibiotic overuse and resistance in hospital settings, as well as the need for more participatory approaches in tackling the complex challenge of AMR. The findings also signify the importance of a bottom-up approach in designing a solution for promoting antimicrobial stewardship and controlling resistance in hospital and community settings. Participatory approaches blended with principles of implementation research will help to identify contextual challenges, and help to design solutions that are people-centred, context-specific and largely accepted by all involved stakeholders.

## Introduction

Excessive consumption of antibiotics is one of the key contributors to the development of antimicrobial resistance (AMR).^1^ In healthcare settings, such practice is largely driven by inappropriate antibiotic prescribing practices by healthcare practitioners, and the lack or non-utilization of treatment guidelines in case management.^2,3^ In resource-limited countries, including Malawi, whose healthcare systems are characterized by a lack of appropriate diagnostic services, empirical antibiotic prescribing is common.^4–6^

Despite limited data on the epidemiology and the burden of AMR due to the scarcity of routine microbiology facilities, it is evident that Malawi shares a heavy burden of AMR.^7,8^ For instance, in 2019, in Malawi, there were 3600 deaths attributable to AMR and 15 700 deaths associated with AMR.^9^ Due to the escalating AMR burden, several initiatives have been established with the aim of combatting the burden. In line with the Global Action Plan (GAP) on AMR, Malawi developed and adopted a corresponding National Action Plan on AMR to guide and coordinate efforts to combat the growing threat of AMR.^10^ The National Action Plan on AMR advocates for education and training of healthcare workers and the use of laboratory data to inform appropriate prescription. However, some studies suggest that awareness-raising and education alone are not sufficient to create meaningful, long-term behaviour change.^11,12^ Effectiveness of education requires communities to take ownership of their information and co-develop solutions that are meaningful in their settings.^13^ This calls for innovative approaches to ensure adequate involvement of stakeholders, along with the communities they serve, to promote sustainability of intervention strategies. Participatory epidemiology (PE) is one of the approaches that could be utilized.

PE, which evolved as a branch of veterinary epidemiology, has been largely utilized for the control and early warning of infectious diseases within resource-limited settings.^14^ It draws on widely accepted techniques of participatory rural appraisal, ethno-veterinary surveys and qualitative epidemiology to engage animal caretakers in identifying and addressing animal health issues, including the design, implementation and evaluation of disease control programmes.^15,16^ PE tools have now expanded to a wider range of applications, like One Health activities to promote community participation, improving people’s understanding of health risks and surveillance options.^16,17^ Since modern epidemiological approaches sometimes fall short of providing complete understanding of situations and finding viable solutions to likely problems relating to community health, PE tools are being employed in human health to derive precise, scientifically credible and contextual information, as well as find sustainable solutions to common community health problems based on local preferences for control options.^18^

Utilizing key local stakeholders in implementing AMR strategies is critical to the success and sustainability of intervention strategies.^19^ In Malawi, antimicrobial stewardship (AMS) teams are potential local stakeholders that could be utilized to promote adoption of interventions addressing AMR.^20^ Moreover, antimicrobial stewardship programmes (ASPs) are essential components of the WHO’s GAP to combat AMR. Effectively implemented ASPs have demonstrated successes in minimizing inappropriate antibiotic usage, and curbing the burden of AMR.^21,22^ However, there are limited data on how they can effectively deliver their roles in hospital settings in resource-limited settings, including in Malawi. Therefore, Malawi’s Antimicrobial Resistance National Coordinating Centre (AMRCC) in collaboration with Clinical Research Education and Management Services (CREAMS) conducted participatory workshops aimed at establishing drivers of AMR and antibiotic overuse in hospitals from the perspective of AMS committees and co-designing facility-friendly intervention strategies against AMR.

The participatory workshops were part of a project jointly implemented by the AMRCC and CREAMS to combat AMR in public health facilities in Malawi. The first phases of the project involved quantitative studies in the public health facilities, evaluating AMR patterns for bloodstream infections and urinary tract infections and evaluating antibiotic prescription practices.

## Methods and procedures

### Study design and setting

**Figure 1.**
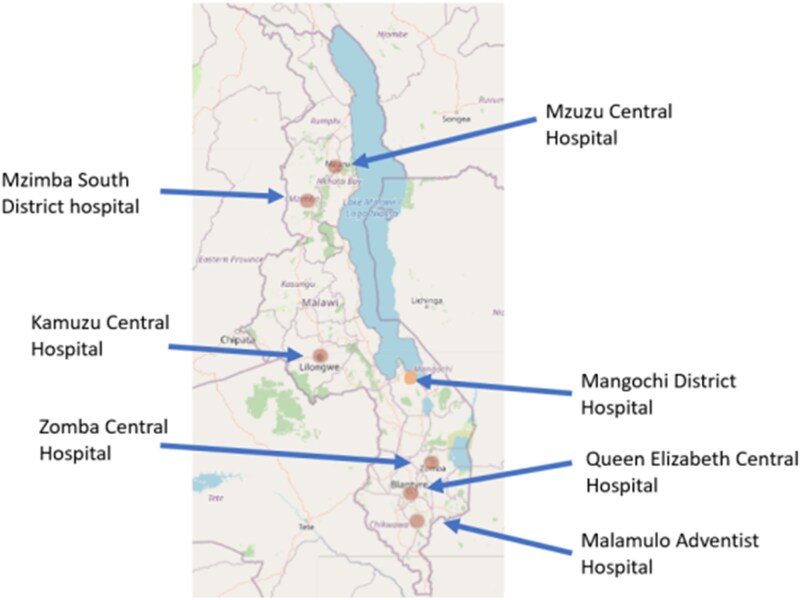
A geographical map of the facilities whose AMS teams were involved in the PE.

The participatory workshops employed qualitative research methods and design thinking exercises to foster discussions among AMS stakeholders. The workshops were conducted across seven public health facilities in Malawi from January 2025 to February 2025 in all three regions of Malawi. Figure 1 represents a geographical map of the facilities whose AMS teams were involved in the PE.

### Recruitment and data collection

Participants were identified using purposive sampling method, recruiting members of hospital AMS teams from the aforementioned health facilities. The exercise was done in three steps, as outlined below.

#### Step 1: Stakeholder mapping

This involved identification of relevant stakeholders involved in the AMS process within the hospital. The AMS committees were made up of committed and collaborative members from all healthcare-related disciplines including clinicians (prescribers), nurses, laboratory personnel, pharmacists and members from quality improvement teams with not less than 2 years’ work experience. Ten AMS members were picked from each facility, projecting the number of participants to 70.

#### Step 2: Information sharing and participatory workshops

This step was conducted between January and February 2025 as a series of participatory workshops held across seven public health facilities in Malawi. The workshops involved sharing current information on antimicrobial use patterns in Malawi and resistance patterns within the hospital setting. The facilitators of the workshops presented point prevalence survey results on antibiotic use at the facility from September 2024 to October 2024, as well as patterns and trends of AMR among commonly isolated pathogens causing bloodstream and urinary tract infections obtained from national microbiological data for the years 2020 to 2024.

The information sharing was intended to bring the participants’ attention to the growing AMR burden and ignite a solution-brainstorming exercise. The information-sharing session was then followed by a participatory discussion to identify challenges associated with AMR control and design solutions. Experienced researchers trained in participatory approaches facilitated the discussion using pre-tested question guides, which were distributed to each of the participants (see Supplementary data, available at *JAC-AMR* Online). Each session was audio-recorded to ensure that all essential data were captured. The sessions were organized in line with the Consolidated Criteria for Reporting Qualitative Research (COREQ).^23^

#### Step 3: Data analysis, report writing and dissemination

After the multi-facility stakeholder workshops, collation of all findings was performed. Four members of the project team listened to all the recordings and transcribed the findings into Microsoft Word. Two other members reviewed the transcripts, having also listened to the recordings three times. The transcripts were then analysed through thematic content analysis to identify common themes and proposed interventions.

### Ethics

Ethical approval to conduct the study was obtained from the National Health Sciences Research Ethics Committee (NHSRC) (Protocol #23/11/4048). Permission to engage the various AMS teams was granted by the hospital directors of the surveyed facilities. Written informed consent was obtained from the participants before the discussions. The whole exercise was conducted in line with ethical principles outlined in the Declaration of Helsinki.

## Results

### Demographic information of participants

A total of 67 key AMS team members participated in the PE workshops across the seven public health facilities. Each team comprised members from various disciplines at the facility. The AMS teams consist of members with not less than 2 years of working at the facility and involvement in AMS activities.

Table 1 shows the breakdown of the participants.

**Table 1.** Characteristics of the study participants

Cadre	Facility							
	QECH	KCH	ZCH	MCH	Mangochi	Mzimba	Malamulo	Total
Clinician	2	1	3	2	4	6	2	20
Nursing officer/NMT	1	2	5	2	2	2	2	16
Lab officer/technician	2	1	1	3	1		3	11
Pharmacist	2		2	1	1	2	1	9
Physiotherapist		1						1
Microbiologist	1							1
Administrator	2							2
Quality manager		2						2
Clerk					1	1		2
IPC/Environmental health officer	1						1	2
Radiologist			1					1
Total	11	7	12	8	9	10	9	67

KCH, Kamuzu Central Hospital; MCH, Mzuzu Central Hospital; NMT, Nurse Midwife Technician; QECH, Queen Elizabeth Central Hospital; ZCH, Zomba Central Hospital.

### Key drivers of antibiotic overuse and resistance patterns

The AMS teams mentioned several factors contributing to inappropriate antibiotic use and AMR in their respective facilities. Following analysis of the discussions, the drivers were classified into the following themes:

#### Policy-related factors

The participants highlighted that lack of or insufficient regulation of policy for access to antibiotics drives overuse of antibiotics. They alluded to antibiotics being easily accessible in private pharmacies and drugstores, where they are usually dispensed without a prescription. Such practices potentially undermine hospital-based efforts to limit usage of antibiotics.

*‘Lack of mandatory guidelines for who gets antibiotics’*—Clinical associate, Zomba

*‘Lack of political will to strengthen policy’*—Medical officer, Mangochi

#### Facility-related factors

One of the key factors that came out was inadequate utilization of facility-based microbiological data on antimicrobial susceptibility patterns to guide prescription practices, which drives poor antibiotic choices for empirical treatment.

‘*Not using the facility-based guidelines that we have to decide who to take cultures on and give treatment. Babies with a fever get ceftriaxone on admission and are discharged the following day on amoxyl. We may not be certain whether they really needed an antibiotic or not’*—Internist, QECH

In addition to that, it was reported that microbiological services testing for antibiotic susceptibility are not always available in most healthcare facilities. As such, several antibiotic classes antibiotics are rolled out, one after another, on a trial basis. This is another key factor contributing to empirical treatment and excessive use of antibiotics.

Another important factor that was highlighted was limited availability of antibiotics in health facilities. Prescribers are compelled to continue using the same antibiotics in excess since more appropriate alternatives are not available. This is due to drug stock-outs, delayed procurement or donor preferences.

‘*We had cefixime, which was donated alongside antiretroviral therapy (ART) drugs, and as such, we just prescribed it because that is what we had available’*—Pharmacy assistant, Mangochi

Additionally, insufficient monitoring of antibiotic prescribing and dispensing practices was also underlined as another key factor driving inappropriate antibiotic use. Most facilities do not use an electronic system for ordering antibiotics. Consequently, anyone could forge prescribers’ notes and provide an easy outlet for antibiotics

‘*Anyone can prescribe an antibiotic by simply writing in the health passport. No one will ask any questions, even if it is a wrong drug.’*—Clinical Associate, Mangochi

Prescribers in private hospitals were also pointed out as contributing to resistance by prioritizing monetary gains by prescribing and dispensing antibiotics for conditions that do not require antibiotic therapy and contrary to treatment guidelines.

‘*The other challenge comes from private facilities, whether hospitals or retail pharmacies; they unnecessarily prescribe antibiotics*—*they dispense antibiotics for profit. The stronger the drug, the more the profit. The solution requires that we find a way to involve them in AMR*’—Nurse, Mangochi

Poor IPC in the facilities was another key factor described to be spearheading the spread of resistant strains of bacteria. Hospital waste is not managed optimally in some facilities, which, coupled with poor hand hygiene practices, predisposes patients and healthcare workers to being at risk of infections.

*‘Poor in-hospital waste management and hand hygiene also contributes to the spread of drug-resistant infections’*—QI, KCH

*‘Sometimes we have no water supply, and we lack soap’*—QI, QECH

#### Healthcare provider-related factors

At times, some healthcare providers prescribe particular antibiotics due to their personal preferences. Such preferences are influenced by the number of doses required to reach therapeutic ranges and ease of administration of the antibiotic.

‘*Watch drugs come as OD doses, which are easier to administer than some Access drugs requiring multiple doses’*—Nurse, KCH

‘*Multiple doses are more difficult to administer in the face of short nursing staff and limited resources such as syringes*’—Nurse, Mangochi

Healthcare providers were also deemed not to adhere to prescription guidelines. In instances that may not necessarily warrant use of an antibiotic, they tend to prescribe one, for fear of missing treatment for an infectious cause to the illness.

‘*We give antibiotics just to cover for a possible infection despite having confirmed the diagnosis’*—Internist, QECH

The aforementioned practice of lack of adherence to treatment guidelines is driven by lack of awareness among some healthcare providers on AMR and their role in propagating or curbing it.

Insufficient patient education during antibiotic dispensing was touted as one of the drivers of inappropriate use of antibiotics. Patient tend to stop taking antibiotics or intentionally skip some doses whenever they feel better, resulting in under-dosage.

*‘Pharmacists not giving enough information to clients, which usually leads to patient stopping taking the drug any time they want’*—Pharmacy assistant, Mangochi

#### Patient-related factors

Inappropriate antibiotic use is also influenced by patients, who have a history of getting better after using a certain antibiotic. As such, when they get a similar or sometimes a different illness, they pressurize the prescribers to give them the same antibiotic. This practice is common among hospital support staff. Some prescribers give in, attributing it to promoting patient satisfaction.

Furthermore, lack of awareness on AMR in the community was reported to result in inappropriate antibiotic use and escalation of AMR. In the communities, outpatients tend to share antibiotics with other members of the community, resulting in consumption of doses below the therapeutic dose.

### AMR resources and perspectives on controlling AMR in hospital settings

Nearly all hospitals recognized the presence of AMS committees, availability of some laboratory point-of-care diagnostics [e.g. full blood count (FBC)], availability of standard treatment guidelines and IPC committees as key resources that help them tackle AMR in the hospital setting. When presented with six proposed interventions for controlling AMR in the hospital setting, the committee ranked the interventions as follows in order of their perceived impact: (i) use of hospital antibiograms to guide prescription practices; (ii) introducing interdepartmental competitions for rational antibiotic use and awards for best practices with regard to AMR control; (iii) having a hospital-based AMR champion to do monthly audits of in-hospital prescription practices and rationale; (iv) monthly continuous professional development training on AMS; (v) use of posters to disseminate messages on rational antibiotic use; and (vi) weekly presentations of hospital AMR profiles.

### Proposed interventions by facilities

Each facility listed several strategies that could be implemented to strengthen their AMS. The design thinking exercise was guided by a conceptual framework adapted from implementation research (IR). The participants designed interventions, bearing in mind that the interventions are influenced by factors from the three context domains of IR, including the outer setting, the inner setting and the stakeholders involved.^24^ The outer setting was defined as the economic, political and social contexts in which an intervention is to be carried out. The outer setting usually cannot be controlled by the implementing hospital or institution. The structure, culture, networks and readiness for change within the implementing hospital constituted the inner setting. The committees were also tasked to identify all stakeholders involved in the control of AMR in the hospital setting. Generally, stakeholders’ knowledge of, attitudes to and perceptions of the intervention and its implementation influences intervention success and impact.^24^ Table 2 summarizes the proposed interventions, along with their context domains for implementation.

**Table 2.** Context domains and stakeholders for the proposed interventions from various AMS teams

Context	Barriers	Facilitators	Proposed intervention
Outer setting			
1. Patients’ need	Patients pressurize clinicians to prescribe specific types of antibiotics if they have worked for them before, even if the illness does not warrant an antibiotic		Capacity building for prescribers to repel patients’ influences in a respectful manner
2. Social setting	Poor awareness of community members on stewardship of antibiotics, which usually led to self-medication		Community awareness on appropriate use of antibiotics Utilizing TV/audio station for public awareness towards AMR
3. External policy	Insufficient regulation of policies on antibiotic use in private pharmacies and drug stores leading to inappropriate antibiotic dispensing	Availability of antibiotic use guidelines in public health facilities	Regular inspection visits to retail pharmacies to enhance adherence to standard prescribing practices Strengthening policies on acquisition of antibiotics in communities
Inner setting			
1. Structure	Microbiological testing services for antibiotic susceptibility are not always available in most health facilities Limited availability of access antibiotics, leading to the overuse of broader-spectrum antibiotics Lack of an electronic system for ordering antibiotics, which potentially results in forging of prescriptions	Existing infrastructure for microbiology services in some facilities	Formulate facility-based drug list and supply as drugs are demanded Facilities to enforce utilization of microbiological laboratory services when treating patients Improve turn-around time for laboratory investigations
2. Culture and networks	Prescribers do not provide adequate education to patient on rational antibiotic use Some prescribers rely on specific antibiotics and are reluctant to be guided by evidence of evolving pathogen susceptibility and resistance patterns during prescribing decisions		Supportive supervision on guideline compliance Introducing ward competition on rational antibiotic use and adherence to guidelines Fostering healthy clinician–client interactions for adequate patient education
3. Available resources	Lack of digital technologies to authenticate prescriptions	Availability of running water for hand hygiene and infection prevention Posters to remind people of the interventions against AMR The multidisciplinary AMS team members enforce measures against AMR in the departments they are based in	Use of digital technologies in ordering and monitoring prescriptions Appointing AMR champions to ensure adequate utilization of all available resources Facilities to ensure adequate supply of resources for infection prevention
4. Access to information		Regular refresher courses to AMS team members on their roles	Sharing antibiograms, audit assessment results on WhatsApp forums Quarterly AMR training for healthcare providers Introducing quarterly continuous professional development (CPD) workshops on AMS
Stakeholders			
1. Individual actors • AMS team members • Clinicians • Pharmacists • Laboratory personnel • Hospital support staff • Community members (patients)	Patients and hospital support staff lacked awareness on rational use of antibiotics and AMR Hospital support staff whose practices were pointed to play a crucial role in spreading AMR were not represented when developing the intervention	All AMS members acknowledged they have an important role to play in promoting awareness about AMR and ensuring adherence to the interventions	Ensuring active involvement of local stakeholders (the AMS teams) in the development of the interventions
2. Organizational actors • AMRCC • Donors • Drug regulatory bodies	Weak enforcement of existing regulations on antibiotic access	AMS members showed willingness to incorporate the interventions in their activities	Regular monitoring of the implementation of interventions Monetary support from donors for AMR initiatives Enforcement of antibiotic access policies in retail pharmacies
Implementation process			
1. Engaging		AMS teams planned to engage community leaders and members on rational antibiotic use by empowering them with knowledge on antibiotic use and AMR Regular training to prescribers to promote adherence to appropriate prescribing standards and utilization of available microbiological testing services	Conduct cross-sectional quantitative and qualitative surveys to understand the extent and drivers of inappropriate antibiotic use at the community level Ensure all essential training on AMR are conducted
2. Evaluating		Regular monitoring and evaluation of the interventions to identify gaps and look for areas requiring improvement	Conduct monthly AMR audits at each facility

## Discussion

To our knowledge, this was the first survey utilizing participatory approaches to identify key drivers for antibiotic misuse and resistance in hospital settings in Malawi, as well as map solutions for tackling the growing burden of AMR. The exercise, which involved discussions, sorting and design thinking exercises, identified limited antibiotic formulary access, cross-sectoral coordination challenges between healthcare, veterinary services, government agencies and private facilities, and culturally specific barriers as unique challenges for AMR control in Malawi. These challenges are embedded in deep health system-related factors, and the cultural and economic fabric of the society.

At the facility level, our study revealed that failure to adhere to guidelines by prescribers, weak enforcement of antibiotic access regulations and insufficient monitoring of antibiotic use were leading contributing factors of AMR. Several studies in sub-Saharan Africa (SSA) have reported the aforementioned drivers to have a massive role in accelerating AMR across Africa.^25,26^ In African settings, health systems are largely constrained, characterized by limited funding, poor infrastructure, weak drug regulatory mechanisms and lack of laboratory diagnostic capacity.^27,28^ As a result, empirical treatment is inevitable. However, it is important for clinicians to adhere to treatment guidelines to minimize antibiotic overuse. Such guidelines should align with local antimicrobial susceptibility profiles of the health facilities. This calls for monitoring of AMR trends and patterns.

Another important challenge that was highlighted was patients demanding specific antibiotics. This practice reflects a culture of self-medication that is prevalent in African settings. In SSA, self-medication prevalence as high as 93% (median prevalence 70%) has been reported, with the commonest source of the antibiotics being community pharmacies.^29,30^ Pressurizing clinicians to provide access to antibiotics is another source of the antibiotics, as our study population alluded to. Similar to our study, the conditions that drive people to self-medicate are usually self-limiting and do not always warrant antibiotic use. Furthermore, community members tend not to complete dosages as they keep some antibiotics to use for similar illnesses in the future or to treat another family member, which further propagates AMR.^31^ Patient education has been shown to increase treatment adherence and reduce improper use of antibiotics.^32^ Therefore, there is a need to sensitize the community to curb patient demands for antibiotics for diseases that do not require antibiotics and the role of improper usage of antibiotics on AMR.

Active involvement of local stakeholders proves key to developing contextually appropriate interventions against AMR. Despite availability of interventions against many health challenges, including AMR, there are many notable gaps in implementation, resulting in less effective interventions.^33^ Several studies, however, have reported that active engagement of local stakeholders in design, implementation, monitoring and evaluation of health interventions leads to effective and sustainable health programmes.^34^ AMS teams, as local stakeholders, act as catalysts for changes, raise awareness among health professionals and the public, and provide leadership to implement AMS interventions.^35^ Therefore, it is important to empower them with knowledge and technical support for coordinating AMS activities in health facilities.

The participatory approach also revealed that the drivers for overuse of antibiotics are impacted by people’s attitude, knowledge, cultural practices and socioeconomic factors. Most people recognized that antibiotic-sharing and acquisition without prescription remains a huge problem and is largely influenced by the socioeconomic status of the people, their demands and the need for more profits. Our current findings are also similar to the previous findings in Malawi and other countries in Africa, which also highlighted patient demands, balance between profit and rational use, and people’s socioeconomic status as key drivers of antibiotic overuse and eventually resistance.^36,37^ For instance, a simulated client study done among community pharmacies in Lilongwe evaluating antibiotic dispensing practices among community retail pharmacies and registered drugstores revealed high rates of non-prescription antibiotic sales, with 53% to 92% of antibiotics dispensed without a prescription for various conditions.^37^ This highlights the influence of the community on antibiotic misuse, which potentially drives resistance.

Similar to other studies, the lack of strict policies and antibiotic dispensing monitoring systems greatly contributes to antimicrobial overuse and eventually resistance. Studies have shown that strict policies are associated with a reduced rate of antibiotic overuse, both in and out of hospital settings. For example, a global policy analysis involving 138 low- to middle-income countries assessing the effectiveness of national policies aimed at reducing antibiotic use and associated mortality due to AMR revealed that regulatory or legislative policies banning over-the-counter sales of antibiotics are associated with a reduction in antibiotic use for lower respiratory tract infections in children. Furthermore, the analysis indicated that stronger AMR governance correlates with reduced total antibiotic consumption at the country level.^38^

Even though the participatory approach revealed notable gaps such as limited human resources, e.g. nurses who drove overuse of easy-to-administer drugs such as ceftriaxone, most of the committees recognized that resources for tackling AMR were generally available. Most importantly, the presence of hospital-based AMS committees was highly recognized as a major win in the fight against AMR in hospital settings. It is evidently clear in the literature that ASPs instigated in hospitals facilitate the prevention of healthcare-acquired and MDR infections.^39^ Multidisciplinary committees involving pharmacy, IPC, quality and performance improvement, medical staff and nursing services remain crucial for effective establishment and operation of ASPs in hospital settings.^40^ In addition, the team also identified other resources such as treatment guidelines, hospital-based AMR posters and IPC posters; however, they cited lack of compliance due to factors such as limited knowledge to be a major hinderance in promoting stewardship of antimicrobials. This is similar to other studies done in SSA, which indicated that in Southern Africa, healthcare professionals’ adherence to evidence-based implementation of standard treatment guidelines for antimicrobial treatment is generally low.^41^

In the design thinking exercise, which was informed by the principles of IR,^24^ participants identified a number of interventions for controlling antibiotic overuse and AMR in hospital settings. Participants centred on interventions that focused on challenges within the hospital setting (inner setting), the people themselves (stakeholders) and the country and policy landscape at large (outer setting). Among others, they advocated for regular training for healthcare workers with regard to AMR, use of digital technologies for ordering, issuing prescription and data management, enhancing strong political will and AMR governance, and the enforcement of antibiotic restriction policies. These were deemed to be some of the actions that would promote rational antibiotic use and lead to reduced antibiotic resistance. Navigating through the key domains of IR, such as the outer setting, the inner setting and identifying individuals involved in spearheading the spread and control of AMR, enabled people to think holistically and focus on dynamic solutions for tackling AMR. The proposed solutions are similar to a participatory study done in Rwanda^42^ highlighting the similarities in challenges faced and the need for more people-centred solutions.

## Strengths and limitations

The use of participatory approaches in our study strengthens the applicability of the interventions formulated. The AMS members put into context the challenges they face and brainstormed solutions that resonate with their local circumstances.

We are aware that this study has some weaknesses. The findings of this study may not be generalizable to the entire population in Malawi. More participatory surveys and design thinking exercises are needed with community members, policymakers and other stakeholders for controlling AMR in low resource settings.

### Conclusions

These findings underscore the complexity of the drivers for antibiotic overuse and resistance in hospital settings, as well as the need for more participatory approaches in tackling complex problems such as AMR. They also highlight the importance of a bottom-up approach in designing solutions for promoting AMS and control of resistance in hospitals, as well as in community settings. Participatory approaches blended with principles of IR will help to identify contextual challenges, and help to design solutions that are people-centred, context-specific and largely accepted by all involved stakeholders.

## Supplementary Material

dlaf103_Supplementary_Data

## Data Availability

The raw data (transcripts) are available upon request.
