# Correction to: Strengthening antimicrobial stewardship in public health facilities in Malawi through a participatory epidemiology approach

**DOI:** 10.1093/jacamr/dlaf173

**Published:** 2025-10-01

This is a correction to: Adriano F Lubanga, Akim N Bwanali, Sibongile Kondowe, Ellen Nzima, Abgail Masi, Yaleka Njikho, Cynthia Chitule, Gracian Harawa, Steward Mudenda, Gillian Mwale, Tumaini Makole, Samuel Mpinganjira, Thomas Nyirenda, Collins Mitambo, Strengthening antimicrobial stewardship in public health facilities in Malawi through a participatory epidemiology approach, *JAC-Antimicrobial Resistance*, Volume 7, Issue 3, June 2025, dlaf103, https://doi.org/10.1093/jacamr/dlaf103

In the originally published version of this manuscript, the following sentences in the ‘Methods and procedures’ section incorrectly stated that the participatory workshops took place in 2024, instead of 2025:

“The workshops were conducted across seven public health facilities in Malawi from January 2024 to February 2024 in all three regions of Malawi.”

“This step was conducted between January and February 2024 as a series of participatory workshops held across seven public health facilities in Malawi.”

This has been corrected and the amended sentences now read as follows:

“The workshops were conducted across seven public health facilities in Malawi from January 2025 to February 2025 in all three regions of Malawi.”

“This step was conducted between January and February 2025 as a series of participatory workshops held across seven public health facilities in Malawi.”

