# Supplementary material for: Strengthening antimicrobial stewardship in public health facilities in Malawi through a participatory epidemiology approach
Source: JAC Antimicrob Resist. 2025 Jun 11;7(3):dlaf103. doi: 10.1093/jacamr/dlaf103 (PMC12152722; doi:10.1093/jacamr/dlaf103)
Supplement: dlaf103_Supplementary_Data [file dlaf103_supplementary_data.pdf]

Participatory Epidemiology Question Guide for Facilitators

Section A: Exploring Drivers of Antibiotic Overuse and Resistance Patterns

1. With regard to the hospital setting in Malawi, what do you think are some of the key drivers of antibiotic overuse and resistance patterns**?** (List as many factors as possible)
2. In your respective field of work, what do you think are the key drivers of Antibiotic overuse and resistance patterns?
3. Across many facilities, there has been a rising trend for antibiotic use from Watch Category in the WHO AWaRE Classification, what do you think are some of the key drivers in your respective facilities.
4. Apart from irrational antibiotic use, what other factors do you think contribute to the growing burden of AMR in Malawi? List as much as you can?

Section 2: AMR Resources and perspectives on controlling AMR in Hospital Settings

5. What resources are available in Malawi to guide rational antibiotic use in hospital setting (These could be policy documents, apps, local guidelines, Posters).
    - Rank the resources according to what you are the most user friendly and effective for promoting steward use of antibiotic in hospital setting
6. Rank the following factors in accordance to their importance in promoting rational use of antibiotics and reducing resistance in hospital settings
    i. Monthly continuous professional developments trainings on antimicrobial stewardship
    ii. Use of hospital antibiograms to guide prescription practices
    iii. Weekly presentations of hospital AMR profiles
    iv. Use of posters to disseminate messages on rational antibiotic use
    v. Having a hospital-based AMR champion to do monthly audits of in hospital prescription practices and rationale
    vi. Introducing interdepartmental competitions for rational antibiotic use and awards for best practices with regard to AMR

Section 3: Design Thinking Exercise; Exploring Barriers and facilitators for AMR mitigation in Hospital settings and Mapping solutions for control

Section Description

copyright@CREAMSmw

The burden of AMR has been escalating in Malawi over the years despite intensification of AMR activities both locally and globally. The national Response has not matched the scale and scope of AMR. Just like in many countries, AMR mitigation efforts face a number of challenges both within and outside the hospital setting. AMR mitigation activities need to be adapted and tailored to local contexts in order to achieve maximum results.

By Utilizing Principles of implementation Research (Figure 1), Explore the potential barriers and Facilitators for AMR control in Hospital Setting and Map solutions for dealing with such.

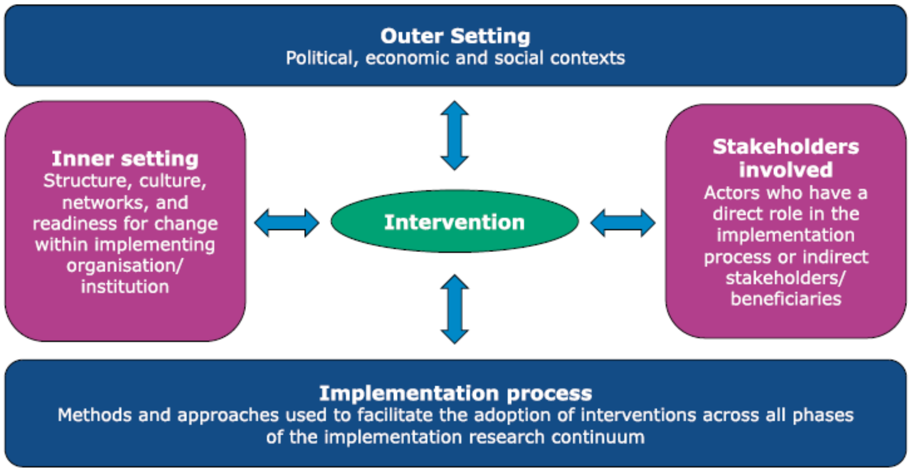

Figure 2. Context domains of IR (adapted from the WHO Implementation Research Toolkit).

Mapping Exercise

   a. How can multidisciplinary teams work together to promote antimicrobial stewardship in hospital setting – (*Please use some illustrations-(diagrams)-think of everyone who works in the hospital? What role do you think each and every person play)-Use illustrated diagrams*

7. What measures have you put in place to

   a. Regulate antibiotic use at
      i. Individual Level
      ii. Departmental level
      iii. Facility level

   b. Addressing antimicrobial resistance as a whole?
      i. Individual Level
      ii. Departmental level
      iii. Facility level

copyright@CREAMSmw
